# Cross-cohort multi-omics analysis identifies novel clusters driven by EMT signatures in colorectal cancer

**DOI:** 10.3389/fimmu.2025.1628005

**Published:** 2025-06-12

**Authors:** Wu Ning, Wenqing Jia, Jingyuan Ning, Lei Zhou, Zongze Li, Lin Zhang, Xin Song

**Affiliations:** ^1^ Department of General Surgery, China-Japan Friendship Hospital, Beijing, China; ^2^ Institute of Clinical Medicine, China-Japan Friendship Hospital, Beijing, China; ^3^ Institute of Basic Medical Sciences, Peking Union Medical College, Chinese Academy of Medical Sciences, Beijing, China

**Keywords:** colorectal cancer, EMT, HOXC6, scRNA-seq, metastatic

## Abstract

**Introduction:**

The pronounced heterogeneity of colorectal cancer (CRC) significantly impacts patient prognosis and therapeutic response, making elucidation of its molecular mechanisms critical for developing precision treatment strategies. This study aimed to systematically characterize tumor cell heterogeneity and explore its clinical implications.

**Methods:**

Five single-cell RNA sequencing cohorts were integrated (comprising 70 CRC samples and 164,173 cells) to systematically analyze tumor cell heterogeneity. Unsupervised clustering analysis based on VEGFR+ tumor cell signature genes was used to stratify CRC patients. Key molecular mechanisms were validated through in vitro cellular experiments, in vivo animal models, molecular docking, and dynamics simulations.

**Results:**

The analysis successfully identified five distinct tumor cell subtypes, with the VEGFR+ subtype exhibiting marked epithelial-mesenchymal transition (EMT) activation signatures and strong association with metastasis and poor clinical outcomes. Based on VEGFR+ signature genes, CRC patients were stratified into three subgroups: C1 (metabolically active), C2 (proliferative), and C3 (invasive), with the C3 subtype demonstrating high metastatic potential, stem-like properties, and an immunosuppressive microenvironment, along with a five-year survival rate below 50%. Mechanistic investigations identified HOXC6 as a key driver of the C3 subtype, with HOXC6 knockout significantly suppressing CRC cell proliferation, migration, and invasion. Furthermore, molecular docking revealed that the targeted agent abemaciclib effectively binds HOXC6, with both cellular and animal experiments confirming its ability to inhibit CRC cell functions and significantly reduce tumor burden in nude mice.

**Discussion:**

This study establishes the first single-cell-resolution molecular classification system for CRC, delineates the mechanistic link between EMT subtypes and metastatic progression, and identifies HOXC6 as a novel therapeutic vulnerability. These findings provide a translational foundation for precision oncology and offer new rationale for precision diagnosis and treatment of colorectal cancer.

## 1 Introduction

Colorectal cancer ranks as the third leading cause of cancer-related mortality worldwide, with five-year survival rates plummeting below 15% following distant metastasis (1). This dire clinical outcome stems from two pivotal biological hallmarks: persistent high recurrence rates and an inherent metastatic propensity observable even at primary tumor stages (2). Current clinical treatment for colorectal cancer relies on 5-FU-based chemotherapy (3), often combined with targeted therapies like anti-EGFR or anti-VEGF agents (4). While these regimens improve response rates, drug resistance and toxicity remain major limitations, especially in advanced-stage disease. Immunotherapy shows promise but is currently limited to a small subset of patients with MSI-H/dMMR tumors, highlighting the need for broader therapeutic strategies (5, 6).

Advances in tumor molecular biology have established tumor heterogeneity as the central driver of these clinical challenges (2, 7). Such heterogeneity manifests not only interpatient variability but, more critically, as spatiotemporal diversity among cellular subpopulations within individual tumors (8). This multilevel complexity fundamentally undermines the efficacy of conventional therapies. The recent advent of single-cell sequencing technologies has revolutionized our capacity to deconstruct tumor heterogeneity at cellular resolution, enabling precise molecular characterization of distinct cellular states within the tumor microenvironment (9).

Within the complex tumor microenvironment, distinct neoplastic subpopulations emerge as pivotal effectors of disease progression through their acquisition of stem-like properties and metastatic competence (10). These metastasis-prone cells are molecularly hallmarked by complete or partial epithelial-to-mesenchymal transition (EMT) activation—a dynamic, reversible biological program that initiates the metastatic cascade through coordinated loss of epithelial polarity and intercellular junctions coupled with gain of mesenchymal traits, including enhanced migratory capacity and apoptosis resistance (11, 12). Notably, recent advances reveal EMT’s pleiotropic role extends beyond physical motility, dually reprogramming cellular metabolism (e.g., augmented glycolysis and glutaminyls) and immune-related molecule expression (e.g., PD-L1 upregulation) to facilitate environmental adaptation during metastatic dissemination (13–15). This process is governed by a multilayered regulatory network: sequential activation of core transcription factors (16) (SNAIL, TWIST, ZEB families (17)) and microenvironmental signals (TGF-β (18), WNT (19), inflammatory cytokines (20)) provide spatiotemporal control. Critically, EMT exhibits marked state plasticity, with tumor cells occupying full, partial, or hybrid EMT states—each demonstrating distinct metastatic potential and therapeutic vulnerability (21). This functional heterogeneity directly contributes to treatment resistance in colorectal cancer. Systematic dissection of EMT’s molecular circuitry therefore offers dual utility: elucidating fundamental principles of metastatic biology while revealing actionable targets for precision therapeutics.

Here we systematically deconstruct colorectal cancer heterogeneity through single-cell transcriptomics, establishing the first EMT activity-based molecular classification that identifies the VEGFR+ EMT subtype—among five distinct tumor cell subpopulations—as clinically associated with metastatic progression and adverse outcomes. Our work mechanistically delineates HOXC6 as a master regulatory node governing this high-risk subtype, with both functional validation and therapeutic targeting experiments confirming its druggable potential. These findings crystallize the central role of EMT reprogramming in metastatic dissemination while providing a framework for precision oncology—from molecular stratification to targeted intervention—that advances personalized therapeutic paradigms in solid tumors.

## 2 Result

### 2.1 Single-cell transcriptomic profiling reveals distinct tumor cell subpopulations in colorectal cancer

**Figure 1:**
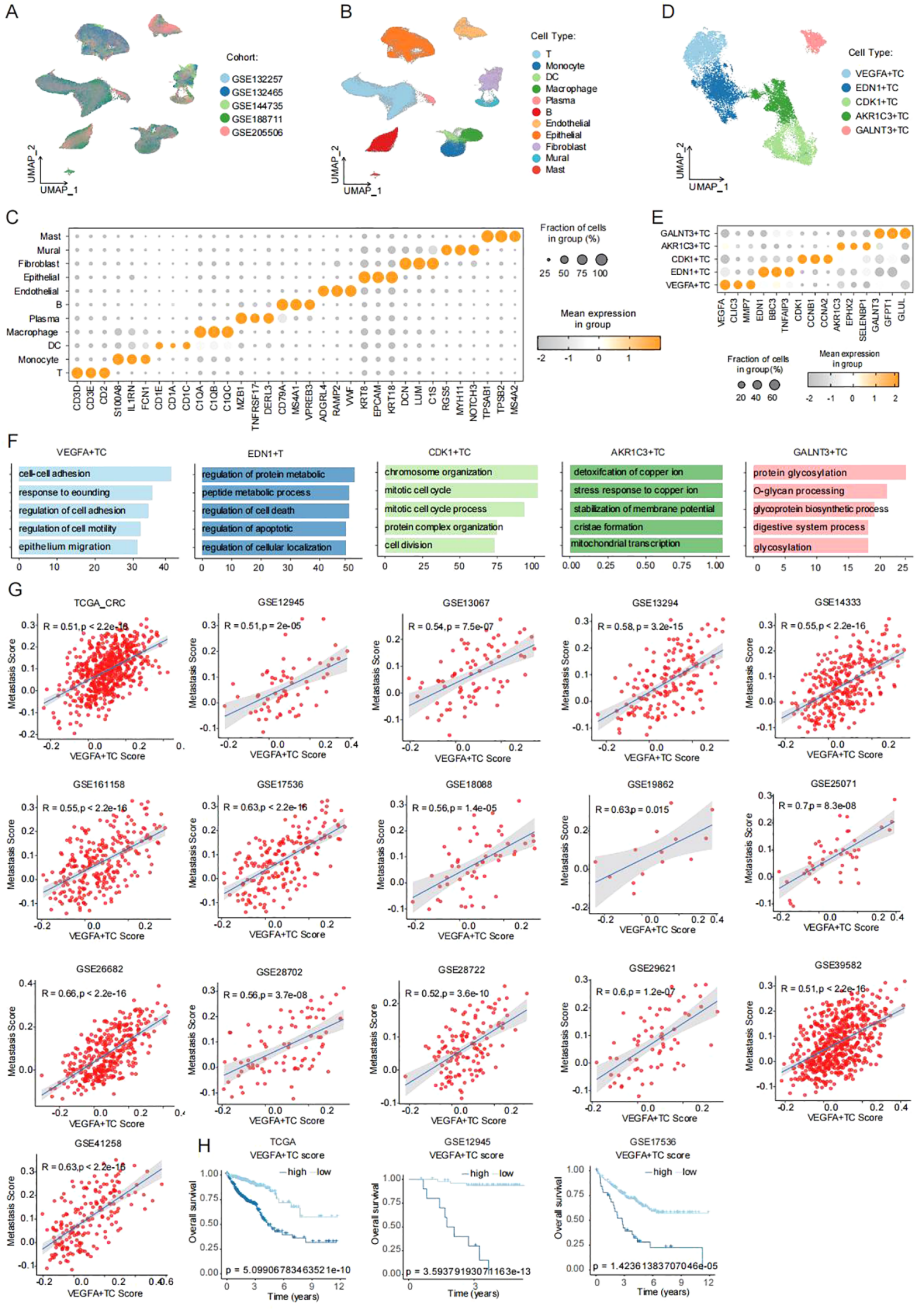
Single-cell sequencing analysis reveals CRC tumor cell heterogeneity **(A)** Dimensionality reduction clustering of five single-cell cohorts. **(B)**UMAP visualization of different cell types. **(C)** Dot plot showing marker gene expression across cell types. **(D)** UMAP visualization of tumor cells. **(E)** Dot plot displays marker genes of five tumor epithelial subtypes. **(F)** Bar plot of enriched pathways from differential gene analysis of five tumor epithelial subtypes. **(G)** Scatter plot showing correlation between VEGFA+TC subtype signature genes and metastatic potential. **(H)** Kaplan-Meier survival curves of patients with high/low VEGFA+TC subtype signature expression across three CRC cohorts.

To systematically characterize tumor cell heterogeneity in colorectal cancer (CRC) patients, we analyzed single-cell RNA sequencing data from five independent cohorts (GSE132257, GSE132465, GSE144735, GSE188711, GSE205506; **Figure 1A**). After stringent quality control, we obtained high-quality transcriptomic profiles for 164,173 cells from 70 CRC samples. Unsupervised clustering based on canonical cell markers identified 11 major cell populations (**Figure 1B**), including plasma cells, B cells, macrophages, dendritic cells, fibroblasts, endothelial cells, mast cells, mural cells, monocytes, T/NK cells, and epithelial cells. The expression patterns of marker genes for each population were visualized using dot plots (**Figure 1C**). For in-depth analysis of tumor cell heterogeneity, epithelial cells were isolated and subjected to further clustering. Malignant cells were identified using the SCEVAN algorithm and subsequently classified into five molecularly distinct subpopulations (**Figure 1D**): VEGFA+TC, EDN1+TC, CDK1+TC, AKR1C3+TC, and GALNT3+TC. Dot plot visualization confirmed the expression of signature genes for each subpopulation (**Figure 1E**). Among these, the VEGFA+TC subpopulation exhibited marked upregulation of CDH1, MMP7, and VEGFA - key regulators of cell migration, invasion, and epithelial-mesenchymal transition (EMT). Functional enrichment analysis of the top 100 differentially expressed genes (log_2_FC > 0.5; adjusted p-value < 0.05) in each subpopulation revealed that VEGFA+TC cells were significantly enriched in pathways related to cell-cell adhesion, cell motility, and epithelial migration (**Figure 1F**), consistent with an EMT phenotype and metastatic potential. The other subpopulations displayed distinct functional characteristics: EDN1+TC was enriched in protein metabolic processes and endoplasmic reticulum stress responses; CDK1+TC showed strong cell cycle and DNA replication activity; AKR1C3+TC was specialized in heavy metal detoxification and oxidative stress response; while GALNT3+TC participated in glycosylation and extracellular matrix remodeling. Validation in bulk transcriptomic datasets (**Figure 1G**) demonstrated a significant positive correlation between the VEGFA+TC signature and metastatic potential. Kaplan-Meier survival analysis (**Figure 1H**) further confirmed that patients with high VEGFA+TC signature expression had significantly worse clinical outcomes. In summary, our single-cell analysis identified a clinically relevant VEGFA+TC subpopulation characterized by EMT activation and heightened metastatic capacity, providing new insights into CRC tumor heterogeneity and progression.

### 2.2 Cross-cohort multi-omics analysis identifies novel clusters driven by VEGFA+TC signatures in colorectal cancer

**Figure 2:**
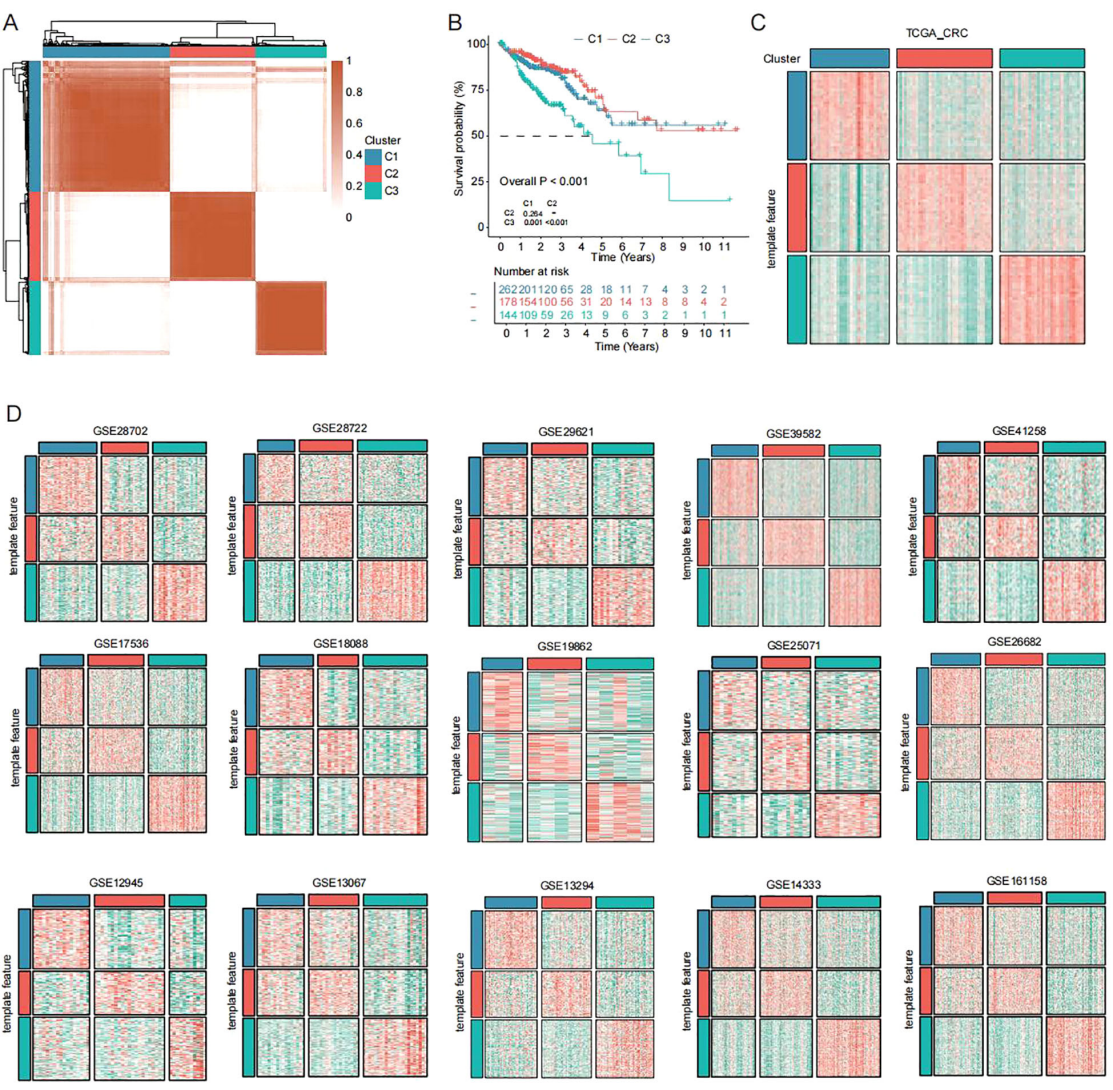
Unsupervised clustering across cohorts identifies new CRC subtypes **(A)** Unsupervised clustering of CRC patients in TCGA dataset based on VEGFA+TC subtype signature scores. **(B)** Kaplan-Meier overall survival curves of three subtypes in TCGA dataset (log-rank test). **(C, D)** Subtype reproducibility across fifteen independent validation cohorts using NTP algorithm.

Using VEGFA+TC signature genes, we performed unsupervised clustering analysis on colorectal cancer patients from the TCGA database. We examined clustering results across different k values (k=2-9). Optimal separation was achieved at k=3 (**Figure 2A**). Kaplan-Meier analysis revealed significant survival differences among the three subtypes, with C3 showing the worst prognosis (**Figure 2B**). The clustering results of the three subtypes remained stable in the TCGA database (**Figure 2C**). Furthermore, we extended the subtype classification using the NTP algorithm. Results demonstrated the stable existence of these three subtypes across multiple cohorts (GSE12945, GSE13067, GSE13294, GSE14333, GSE161158, GSE17536, GSE18088, GSE19862, GSE25071, GSE26682, GSE28702, GSE28722, GSE29621, GSE39582, GSE41258) (**Figure 2D**).

### 2.3 Significant differences in biological functions among the three subtypes

**Figure 3:**
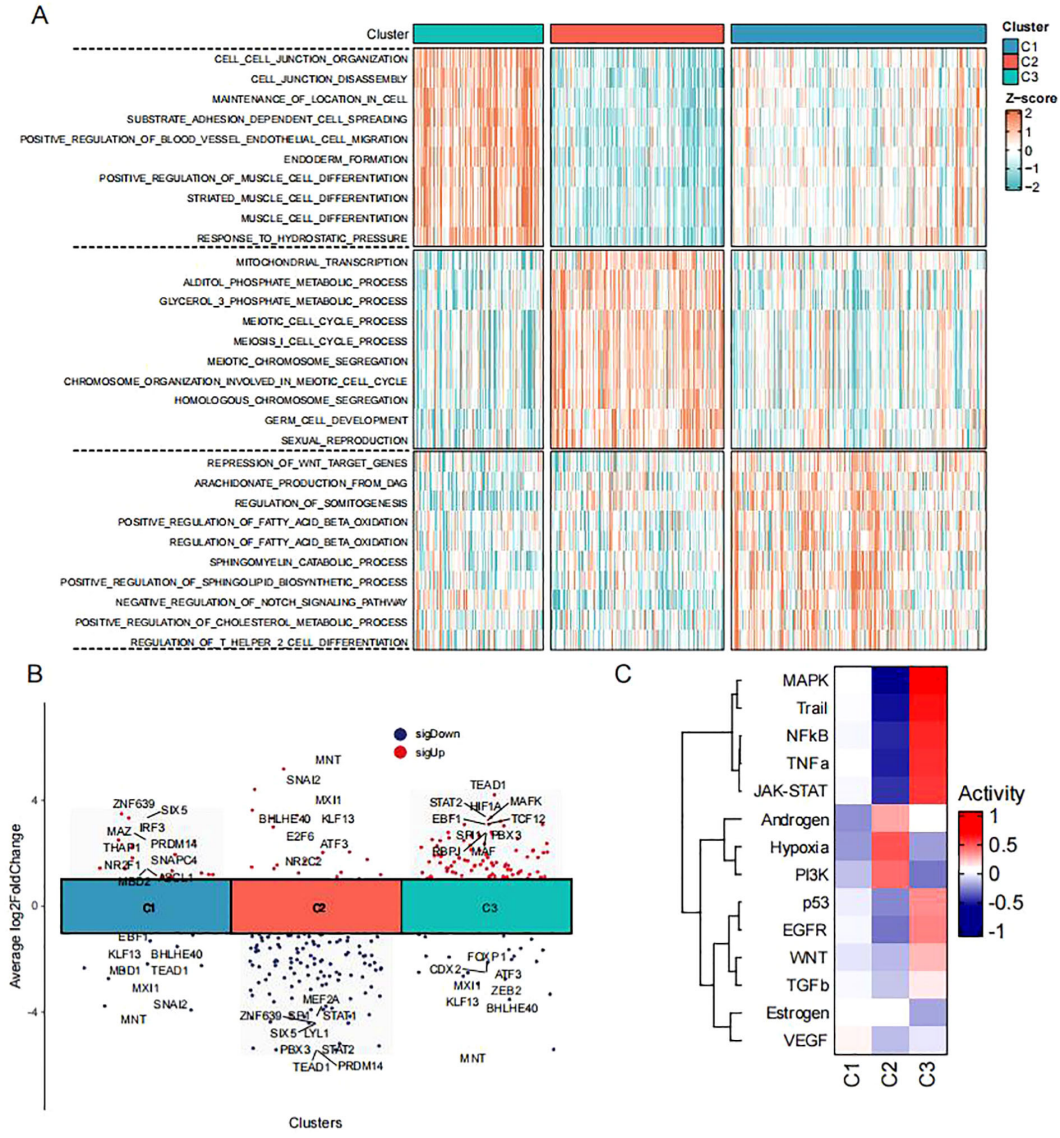
Functional pathway alterations in CRC subtypes **(A)** Heatmap of biological processes across three subtypes in TCGA-CRC dataset (red/blue indicate high/low ssGSEA scores). **(B)** Volcano plot showing differential transcription factor activity analysis among three subtypes in TCGA-CRC dataset. **(C)** Heatmap of pathway activation states across three subtypes in TCGA-CRC dataset.

To characterize the biological behavior of these subtypes, we calculated subtype-specific biological processes using ssGSEA (**Figure 3A**). Subtype C1 exhibited suppression of Wnt target genes, along with enrichment in lipid metabolism (arachidonic acid production, sphingolipid catabolism) and immune regulation (negative regulation of Notch signaling, regulation of Th2 cell differentiation). Subtype C2 was associated with mitochondrial transcription and metabolic processes (alditol phosphate metabolism, glycerol-3-phosphate metabolism), as well as meiotic cell cycle and germ cell development, suggesting connections to reproductive biology. Subtype C3 showed enrichment in cell junction organization and adhesion-dependent processes (matrix adhesion-dependent cell spreading, positive regulation of endothelial cell migration), indicating a role in cell motility. We next quantified transcription factor activity across the three subtypes (**Figure 3B**). Results showed subtype C1 highly expressed transcription factors including ZNF639, SIX5, IRF3, and MAZ, suggesting associations with development and metabolism. Subtype C2 showed high expression of MNT, SNAI2, and MXI1, indicating associations with cell proliferation and cell cycle. Subtype C3 exhibited high expression of TEAD1, STAT2, HIF1A, MAFK, EBF1, and TCF12, suggesting involvement in cell migration. Additionally, PROGENy analysis (**Figure 3C**) revealed significant activation of MAPK, NF-κB and TNFα signaling pathways in subtype C3, while hypoxia and PI3K pathways were suppressed. This suggests subtype C3 may acquire metastatic advantages by remodeling signaling networks - utilizing inflammatory pathways (NF-κB/TNFα) to drive invasion while downregulating metabolism-adaptation pathways (PI3K) to maintain energy balance during metastasis. This unique “inflammatory activation-metabolic suppression” pattern further supports the highly metastatic nature of subtype C3.

### 2.4 Immunological landscape of the three subtypes

**Figure 4:**
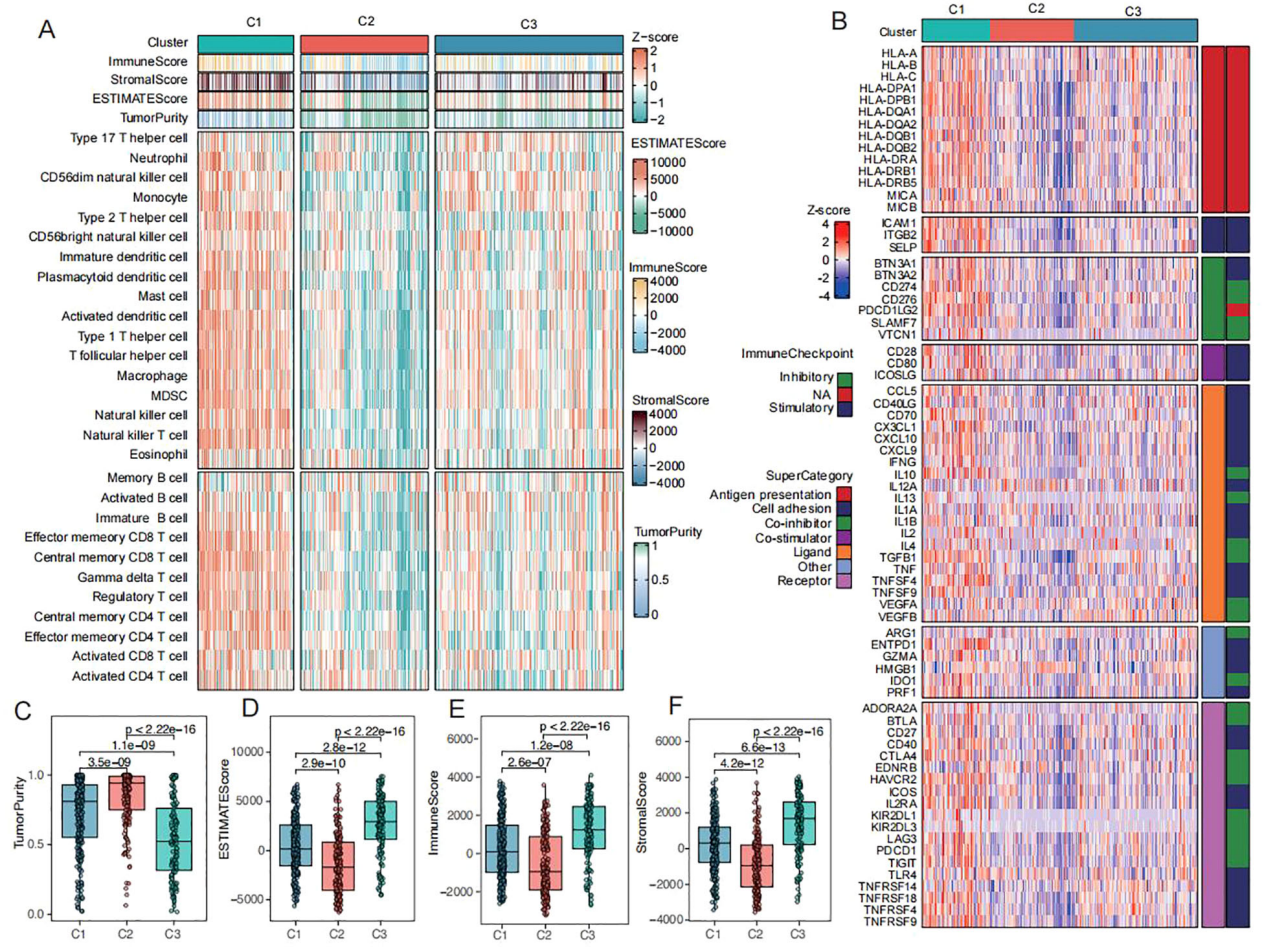
Immunization patterns of CRC subtypes **(A)** Heatmap of immune cell infiltration and immune-related pathways across three subtypes in TCGA-CRC dataset (red/blue indicate high/low ssGSEA scores). **(B)** Heatmap of antigen presentation, co-stimulatory/co-inhibitory molecules, immune checkpoints, and cytokine receptor-ligand expression across three subtypes. **(C-F)** Comparisons of tumor purity **(C)**, ESTIMATE scores **(D)**, immune scores **(E)**, and stromal scores **(F)** among three subtypes in TCGA-CRC dataset.

Our study systematically characterized the heterogeneity of immune microenvironments across the three CRC subtypes (C1-C3). Quantitative analysis of immune cell infiltration using ssGSEA revealed that the C3 subtype exhibited the most extensive immune cell infiltration, including key antitumor effector populations such as activated CD8+ T cells and CD4+ T cells (**Figure 4A**). Notably, this subtype concurrently overexpressed multiple immunomodulatory molecules, including antigen-presenting proteins (HLA-A/B/C), co-stimulatory molecules (CD28/CD80), and immune checkpoint proteins (PD-L1) (**Figure 4B**). However, the simultaneous enrichment of regulatory T cells, neutrophils, and monocyte-derived macrophages established a characteristic “hot but suppressed” immune microenvironment in C3. ESTIMATE algorithm analysis further confirmed these findings (**Figures 4C-F**), demonstrating that C3 possessed the highest immune and stromal scores but the lowest tumor purity. In contrast, the C2 subtype displayed a typical “immune-desert” phenotype with maximal tumor purity and minimal immune infiltration, while C1 exhibited transitional immune characteristics. These results not only validate the complexity of CRC immune microenvironments but, more importantly, reveal the unique coexistence of immune activation and suppression mechanisms in the C3 subtype. This finding provides novel insights into the biological basis of immunotherapy resistance in colorectal cancer.

### 2.5 HOXC6 serves as a marker molecule for C3 and correlates with poor prognosis

**Figure 5:**
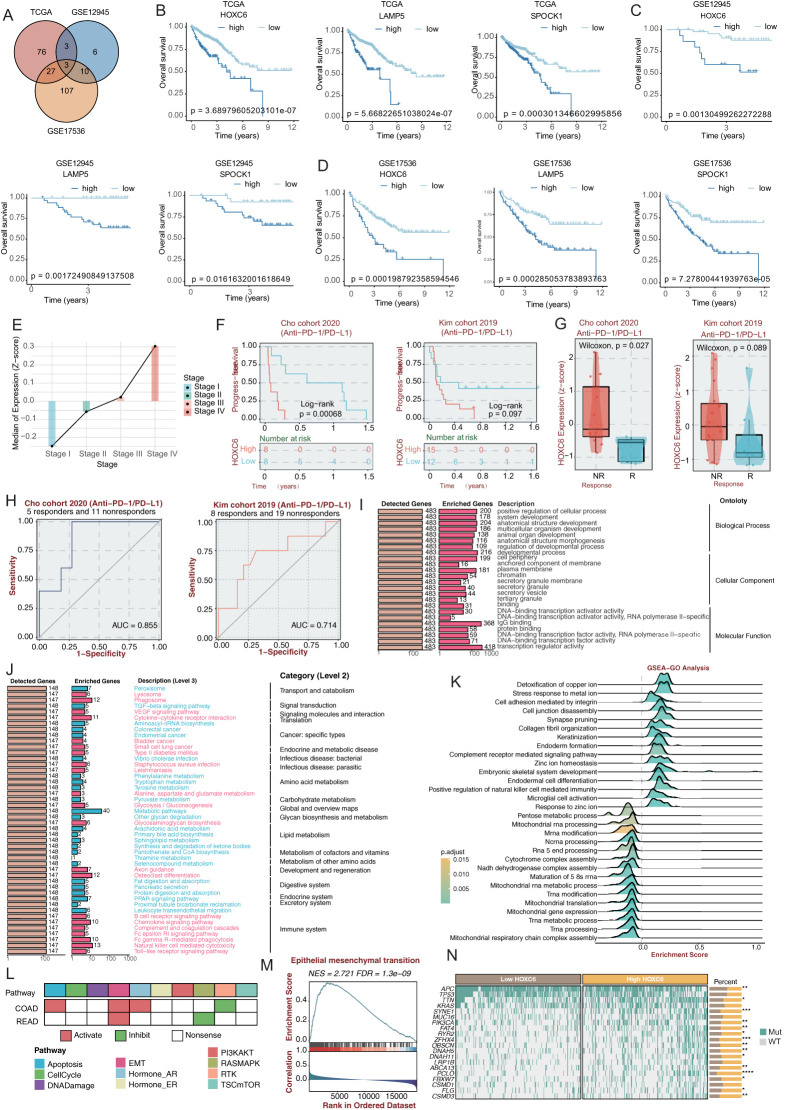
HOXC6 promotes poor prognosis in CRC patients **(A)** Venn diagram of prognosis-associated genes across three cohorts. **(B)** Kaplan-Meier overall survival curves for HOXC6, LAMP5 and SPOCK1 in TCGA-CRC and two validation cohorts (log-rank test). **(C)** Association between HOXC6 expression and CRC clinical stages. **(D)** Progression-free survival in anti-PD-1/PD-L1 treated patients stratified by HOXC6 expression. **(E)** ROC analysis of HOXC6 expression for predicting immunotherapy response (AUC values shown). **(F)** GO functional analysis of HOXC6-associated biological processes. **(G)** KEGG pathway enrichment analysis. **(H)** GSEA pathway activation analysis. **(I)** Biological processes associated with HOXC6 in CRC. **(J)** Correlation between HOXC6 and EMT pathway in CRC. **(K)** Genomic analysis of HOXC6 expression and mutation profiles. **(L)** Pathway activity analysis of patients with high HOXC6 expression. **(M)** GSEA analysis of EMT pathway in patients with high HOXC6 expression. **(N)** Correlation analysis between HOXC6 expression level and gene mutation status.

To elucidate the molecular characteristics and clinical significance of the C3 subtype with the worst prognosis, we identified three genes (HOXC6, LAMP5, and SPOCK1) through intersection analysis of C3 signature genes across TCGA, GSE12945, and GSE17536 cohorts (**Figure 5A**). Subsequent survival analyses consistently identified HOXC6, LAMP5, and SPOCK1 as significant prognostic risk factors. Kaplan-Meier analysis demonstrated significantly shorter median overall survival in patients with high expression of these genes (**Figure 5B**). The homeobox (HOX) genes encode a group of transcription factors that bind DNA and regulate gene expression, functioning as either activators or repressors (22, 23). In cancer biology, these genes frequently exhibit mutations or elevated expression levels, where they actively participate in driving malignant transformation and tumor advancement (24). Leveraging powerful platform, BEST, we conducted an in-depth analysis of HOXC6’s functional expression patterns across diverse cancer types. The database’s robust datasets enabled us to elucidate HOXC6’s prognostic significance and potential biological roles in CRC (25).Clinical staging analysis showed a progressive increase in HOXC6 expression levels with disease advancement (**Figure 5E**). Analysis of anti-PD-1/PD-L1 treated patient cohorts revealed superior progression-free survival in the HOXC6 low-expression group (**Figures 5F-G**). Receiver operating characteristic (ROC) curve analysis demonstrated that HOXC6 expression levels achieved area under the curve values of 0.855 and 0.714 in two independent validation cohorts for predicting immunotherapy response (**Figure 5H**), indicating significant predictive value. Functional enrichment analysis showed HOXC6-associated genes significantly enriched in fundamental biological processes including DNA binding and secretory system regulation, particularly transcriptional activation and vesicular transport functions (**Figure 5I**). KEGG pathway analysis revealed HOXC6 primarily activates signal transduction pathways, including VEGF signaling and cytokine receptor interaction pathways (**Figure 5J**). GSEA pathway enrichment demonstrated HOXC6’s association with cell junction assembly and cell adhesion (**Figure 5K**), findings highly consistent with C3 subtype characteristics and forming a comprehensive functional network from cell junction assembly to membrane skeleton formation. Notably, HOXC6 showed strong correlation with epithelial-mesenchymal transition (EMT) programs (**Figure 5L**). In colorectal cancer, the HOXC6 high-expression group exhibited typical EMT features, including downregulation of epithelial markers and upregulation of mesenchymal markers (**Figure 5M**). Genomic analysis revealed significant correlations between HOXC6 expression and specific mutation profiles, with APC and TP53 mutations more prevalent in the low-expression group (**Figure 5N**). This suggests HOXC6’s potential role in regulating genomic stability, consistent with the loss of function of the APC tumor suppressor in CRC, which is associated with poor prognosis (26). AND the loss of function of TP53 was reported to lead to the formation of an NF-κB dependent inflammatory microenvironment and triggers EMT (27). These findings collectively establish HOXC6 as a key regulatory molecule for the C3 subtype, demonstrating not only prognostic value but also providing a potential precision therapeutic target for this patient subgroup.

### 2.6 HOXC6 knockdown suppresses malignant behaviors of colorectal cancer cells *in vitro* and *in vivo*

**Figure 6:**
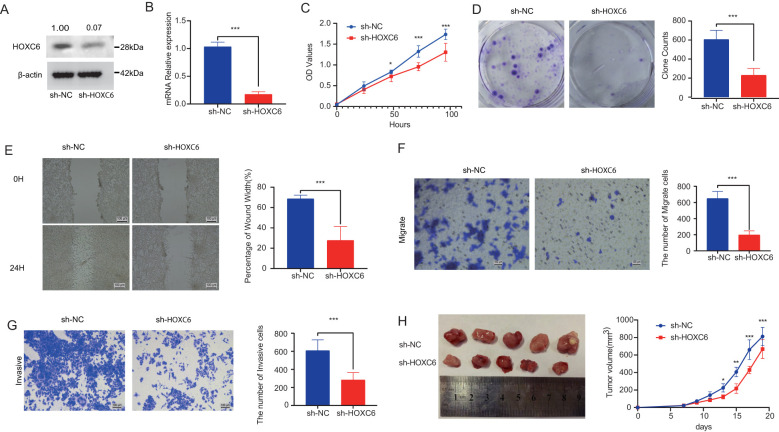
HOXC6 promotes cell proliferation and invasion in *in vivo* and *in vitro* assays **(A)** HOXC6 protein expression in sh-HOXC6 *vs* sh-NC groups. **(B)** Relative HOXC6 mRNA levels. **(C)** CCK-8 assay OD450 values. **(D)** Colony formation capacity. **(E)** Wound healing rates at 24h. **(F)** Number of migrated cells. **(G)** Number of invaded cells. **(H)** Tumor sizes in mouse xenografts.

To investigate the effects of HOXC6 on colorectal cancer cell behaviors including proliferation, migration, and invasion, we established HOXC6-knockdown colorectal cancer cell lines. HOXC6 mRNA expression was significantly reduced in these cell lines (**Figure 6A**). CCK-8 assays performed 24 hours post-transfection demonstrated that the proliferation rate was significantly decreased in the sh-HOXC6 group compared to the sh-NC group (**Figure 6C**). Similarly, colony formation assays revealed a marked reduction in clonogenic potential in the sh-HOXC6 group relative to the sh-NC group (**Figure 6D**). These findings collectively indicate that HOXC6 knockdown suppresses colorectal cancer cell proliferation. Wound healing assays (**Figure 6E**) and Trans-well migration assays (**Figure 6F**) showed that the migration rate was significantly lower in sh-HOXC6 cells compared to sh-NC cells, demonstrating that HOXC6 downregulation inhibits colorectal cancer cell migratory capacity. Furthermore, Trans-well invasion assays conducted with these cell lines (**Figure 6G**) revealed a significant decrease in the number of cells penetrating through the membrane in the sh-HOXC6 group versus the sh-NC group, indicating that HOXC6 knockdown impairs the invasive capability of colorectal cancer cells. Quantitative analysis of tumor volumes at day 20 post subcutaneous implantation showed significantly reduced tumor sizes in the sh-HOXC6 group compared to the sh-NC group.

### 2.7 Abemaciclib targets HOXC6 and effectively inhibits tumor proliferation

**Figure 7:**
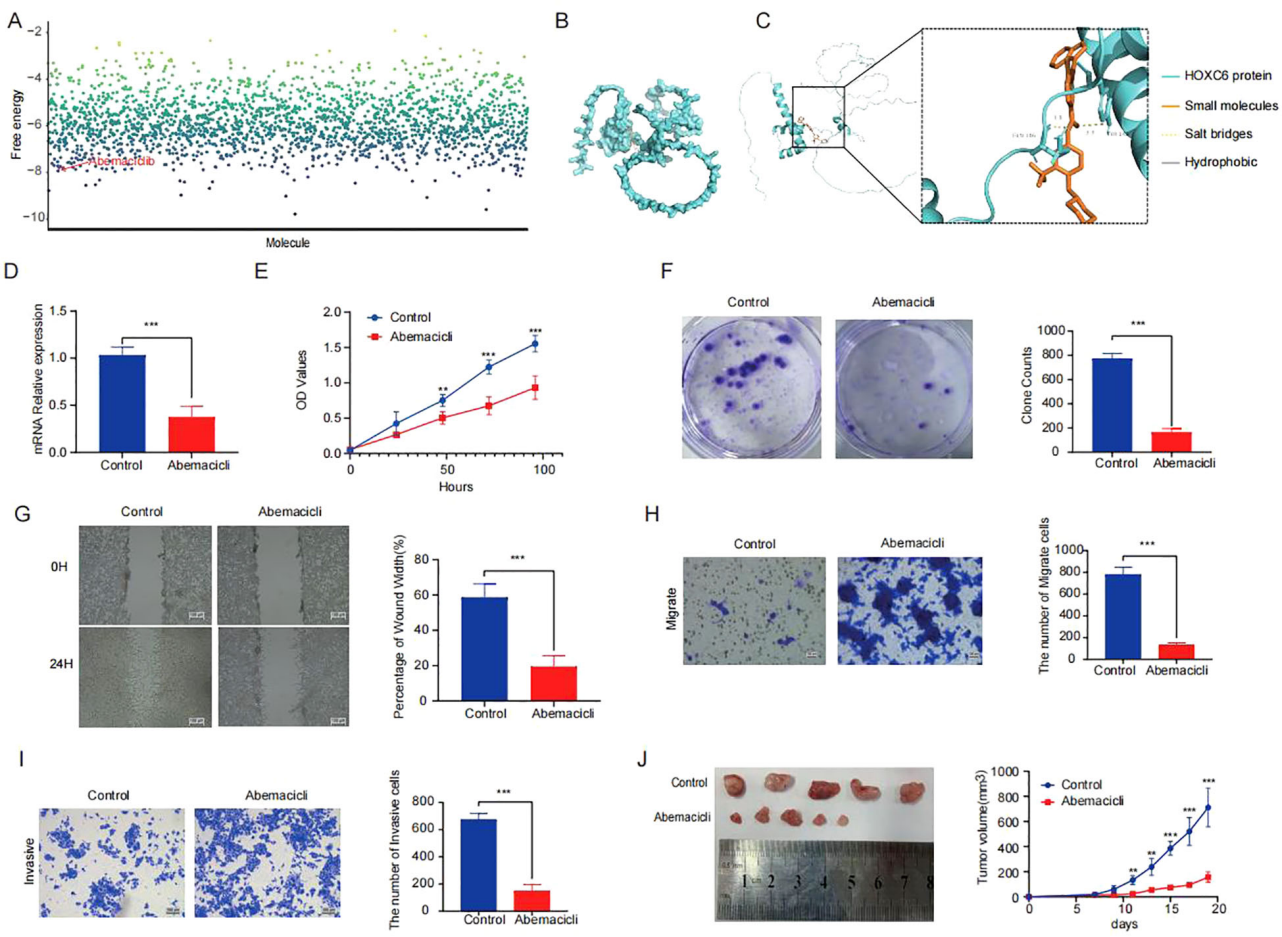
Abemaciclib stabilizes the targeting of HOXC6 **(A)** Binding free energy analysis from molecular docking of HOXC6 with drug candidates. **(B-C)** Interaction diagrams of abemaciclib within HOXC6 binding pocket. **(D)** Relative HOXC6 mRNA levels in abemaciclib *vs* control groups. **(E)** CCK-8 assay OD450 values. **(F)** Wound healing rates at 24h. **(G)** Number of migrated cells. **(H)** Number of invaded cells. **(I)** Colony formation capacity. **(J)** Tumor sizes in mouse xenografts.

Through an integrated approach combining computational drug screening and experimental validation, we systematically evaluated the therapeutic potential of HOXC6 in colorectal cancer. Initial virtual screening of 2,110 FDA-approved compounds using molecular docking identified abemaciclib as exhibiting optimal binding characteristics based on free energy calculations (**Figure 7A**). Detailed docking analysis revealed that abemaciclib forms a stable interaction network within the HOXC6 active pocket, including hydrogen bonds with TYR-148 and GLN-146 residues (**Figures 7B-C**). *In vitro* functional assays confirmed abemaciclib’s therapeutic potential. Treatment with abemaciclib significantly downregulated HOXC6 mRNA expression in colorectal cancer cell lines (**Figure 7D**). Systematic cellular functional experiments demonstrated abemaciclib’s targeted inhibition of HOXC6 and its antitumor effects. CCK-8 assays showed significantly reduced cell proliferation 24 hours post-treatment (**Figure 7E**), while colony formation assays (**Figure 7F**) confirmed abemaciclib’s ability to markedly decrease colony numbers, indicating effective inhibition of tumor cell proliferation through HOXC6 targeting. Migration capacity assessments, including wound healing (**Figure 7G**), Transwell migration (**Figure 7H**), and invasion assays (**Figure 7I**), consistently demonstrated significantly reduced cell migration rates and membrane-penetrating cell numbers in abemaciclib-treated groups, confirming the drug’s ability to effectively block HOXC6-mediated metastatic potential. In nude mouse subcutaneous tumor models (**Figure 7J**), abemaciclib-treated groups showed significantly smaller tumor volumes compared to controls. These multi-level results, from molecular interactions to cellular phenotypes, collectively demonstrate that abemaciclib specifically targets HOXC6, forms stable complexes, and significantly inhibits colorectal cancer cell proliferation, migration, and invasion capabilities.

## 3 Discussion

Colorectal cancer, as one of the most heterogeneous malignancies in the digestive system, presents significant clinical challenges due to variations in treatment response and prognosis (28). CRC pathogenesis stems from the dysregulation of complex signaling networks that orchestrate key cellular functions such as proliferation, survival, and apoptosis (29). This aberrant signaling leads to the malignant transformation of the colonic epithelium. This study systematically characterized CRC tumor cell heterogeneity through single-cell RNA sequencing, leading to the first identification of the VEGFA+TC subpopulation with EMT activation and high metastatic potential. Based on its molecular features, we classified CRC patients into three stable subtypes. This classification demonstrated high reproducibility across multiple independent cohorts (TCGA and various GEO datasets), with the C3 subtype showing the highest EMT activity and poorest clinical outcomes, highlighting the clinical predictive value of this stratification system.

Multi-omics data and functional experiments systematically revealed the central regulatory role of HOXC6 in specific CRC subtypes, providing novel insights into tumor heterogeneity. Our findings demonstrate that HOXC6 not only serves as a marker molecule for the aggressive C3 subtype, but also drives disease progression through multiple mechanisms including EMT program regulation, metabolic reprogramming, and immune microenvironment remodeling. Previous study identified TGF-β signaling exhibiting a dual role in CRC by suppressing early tumorigenesis but promoting EMT-driven metastasis in advanced stages through epigenetic dysregulation of its pathway components (30). Validation studies confirmed that HOXC6 is closely associated with EMT pathway activation and directly regulates CRC cell proliferation, migration, and invasion capabilities. Both *in vitro* and *in vivo* functional experiments showed that HOXC6 knockdown significantly inhibited tumor growth and metastasis, confirming its oncogenic role. Additionally, patients with high HOXC6 expression showed poorer response to PD-1 inhibitors, while those with low HOXC6 expression exhibited higher APC/TP53 mutation burden, suggesting HOXC6 may promote tumor progression through mutation-independent transcriptional regulatory mechanisms. These findings not only fill the knowledge gap regarding molecular mechanisms of EMT and immune microenvironment crosstalk, but also provide novel biomarkers (e.g., HOXC6) and potential therapeutic targets for precision classification and targeted intervention in CRC.

Although the role of HOX genes in immunotherapy resistance has not been directly studied, their involvement in anti-angiogenic therapy resistance suggests a possible connection. HOX genes, such as HOXB9, promote tumor aggressiveness by upregulating pro-angiogenic and pro-inflammatory factors, which can also contribute to an immunosuppressive microenvironment (31). Since inflammation and myeloid cell recruitment are key mechanisms of resistance to both anti-angiogenic and immunotherapies, targeting HOX genes may help overcome treatment resistance. Functional studies further suggested that elevated expression of HOXB13 is related to ER downregulation and, consequently, to TAM-resistance in ER+ cancers (32).Further research should explore whether HOX family members influence immune evasion and response to checkpoint inhibitors.

Research across multiple cancer types has established that aberrant overexpression of transcription factors serves as a key driver of both primary tumor growth and distant metastasis (33–35). The homeobox protein HOXC6 represents one such factor that exhibits cancer-specific upregulation and functionally contributes to malignant cell expansion. HOXC6 promotes cervical cancer progression by transcriptionally upregulating BCL2 to enhance anti-apoptotic effects and drive tumor cell proliferation (36) In prostate cancer, HOXC6 promotes tumor cell survival by transcriptionally repressing proapoptotic factors NEP and IGFBP-3, and its knockdown induces caspase-dependent apoptosis in both androgen-dependent and -independent cancer cells (37). However, in CRC, key questions remain regarding HOXC6’s expression patterns, regulatory networks, and mechanistic contributions to EMT. Our single-cell resolution study not only confirms HOXC6’s conserved oncogenic role across tumors but also establishes EMT’s pivotal position in CRC metastasis. Elucidating HOXC6’s EMT-regulatory mechanisms will advance understanding of tumor heterogeneity and may yield novel molecular classification systems and therapeutic targets for precision medicine.

The discovery of abemaciclib’s activity against HOXC6 represents a breakthrough in targeting transcription factors (TFs), traditionally considered “undruggable” due to their lack of defined binding pockets (38, 39). Our molecular dynamics simulations first identified an allosteric site within HOXC6’s DNA-binding domain. Mechanistically, abemaciclib stabilizes HOXC6’s inactive conformation, disrupting coactivator recruitment. Abemaciclib is an oral CDK4/6 inhibitor with preferential activity against CDK4 (40). Its favorable safety profile, including lower rates of myelosuppression, permits continuous dosing unlike other approved agents in this class (41, 42). Emerging preclinical synergy between cell cycle inhibitors (CDK4/6i) and either PI3K pathway blockers or immunotherapies is now being translated into clinical trial designs (43).Our studies revealed that while HOXC6-high tumors respond poorly to PD-1 inhibitors, they exhibit marked sensitivity to abemaciclib—potentially through drug-mediated immune microenvironment remodeling. Immune checkpoint inhibitors targeting the PD-1/PD-L1 axis have gained monotherapy approval for advanced colorectal cancers exhibiting mismatch repair deficiency (dMMR) and/or high microsatellite instability (MSI-H) (44–46). For high-risk C3-subtype patients, combination strategies incorporating HOXC6 targeting may be essential. Thus, combining Abemaciclib with immunotherapy could improve treatment sensitivity in HOXC6-high colorectal cancer patients.

In conclusion, the C3 subtype represents a clinically relevant EMT population whose characterization advances CRC heterogeneity understanding and precision therapy development. Future efforts should focus on translating these discoveries into clinical practice to improve outcomes. Our integrated computational-structural-preclinical approach not only establishes HOXC6 as a novel therapeutic target but also pioneers innovative TF-targeting paradigms, potentially ushering in a new era of molecular subtype-directed CRC treatment.

## 4 Methods

### 4.1 Data collection

For this study, we obtained 15 colorectal cancer cohort datasets from The Cancer Genome Atlas (TCGA) and Gene Expression Omnibus (GEO) databases (GSE12945, GSE13067, GSE13294, GSE14333, GSE161158, GSE17536, GSE18088, GSE19862, GSE25071, GSE26682, GSE28702, GSE28722, GSE29621, GSE39582, and GSE41258). The RNA-seq count data from TCGA-CRC were converted to transcripts per million (TPM) and subsequently log2-transformed. Each dataset was matched with its respective sequencing platform. In cases where a gene was represented by multiple probes, the gene expression values were averaged. Subsequently, data were normalized in R (version 4.4.0) using the “normalizeBetweenArrays” function from the “limma” package (version 3.48.1). Each dataset was analyzed independently, thereby mitigating batch effects.

### 4.2 Single-cell RNA sequencing data analysis

Initial data processing was performed using Seurat (v4.0.4), where raw data from five CRC single-cell cohorts were imported via the Read10X function and converted to sparse matrix format (dgCMatrix). Datasets were integrated using the merge function with unique cell identifiers ensured by RenameCells. Quality control included: 1) Scrublet-based doublet removal; 2) filtration of low-quality cells (<100 genes detected); 3) exclusion of genes expressed in <3 cells. Data normalization employed LogNormalize scaling (factor=10,000) with identification of top 2,000 highly variable genes. Technical variation was regressed out using ScaleData (covariates: UMI counts and mitochondrial gene content). Dimensionality reduction (top 30 PCs) was followed by Harmony-based batch correction (47). UMAP visualization and Louvain clustering (resolution=0.6) were performed, with optimal clustering determined via clustree. Cell type annotation utilized established marker genes and literature references. Epithelial subpopulations were re-analyzed through repeated dimensionality reduction/clustering (resolution=0.2), with functional annotation based on differentially expressed genes.

### 4.3 Functional annotation analysis

Functional annotation analysis was performed using ClusterProfiler (v4.4.4) (48). The analysis workflow comprised: (1) gene ID conversion from Ensembl IDs to standard gene names via the bitr function; (2) Gene Ontology (GO) enrichment analysis using enrichGO, covering Biological Process (BP), Molecular Function (MF), and Cellular Component (CC) categories; (3) KEGG pathway analysis via enrichKEGG. Statistical significance was defined as P<0.05 with Benjamini-Hochberg multiple testing correction applied to all results.

### 4.4 Unsupervised clustering

To elucidate tumor heterogeneity in gastric cancer patients, we employed a sample clustering method. The clustering was based on the signature gene of the VEGFR+TC subtypes. Specifically, sample clustering analysis was conducted using the ConsensusClusterPlus (49)package in R. The maximum number of clusters (K) was set to 9, with a step size of 1, to evaluate clustering results at different K values. To ensure the robustness of the clustering results, the clustering analysis was repeated 1,000 times. Pltem was used as a feature retention threshold, determining the number of features (genes) retained for clustering analysis in each bootstrap iteration. In our setup, Pltem was set to 0.8, meaning that 80% of the features were retained in each bootstrap iteration. The pFeature parameter indicated the probability of feature retention, determining whether each feature would be retained in each bootstrap iteration. In this case, pFeature was set to 1, implying that all features were retained without feature selection. This setup is suitable for scenarios where all features need to be considered, or when the number of features is relatively small. The clustering process employed the k-means clustering algorithm (clusterAlg = “km”) and Euclidean distance (distance = “Euclidean”). The results were visualized using the “ggplot2” package to assess potential algorithmic biases in the ConsensusClusterPlus clustering method.

### 4.5 Single-sample gene set enrichment analysis

We used the ssGSEA algorithm to study the enrichment of differential gene sets across different samples. ssGSEA is a computational method designed for single-sample gene set enrichment analysis, providing insights into the activity levels of differential genes in individual samples. The differential gene sets used in this study were derived from research by the Alexander Bagaev team. We implemented ssGSEA using the “GSVA” package in R and the “gsva()” function(Bagaev et al., 2021). In this analysis, the method parameter was set to “ssgsea,” and enrichment analysis was performed using Gaussian kernel density estimation (kcdf = “Gaussian”). Additionally, we retained the absolute ranking information (abs.ranking = TRUE). To normalize the ssGSEA results, the ssGSEA scores were scaled to a range of 0 to 1, which facilitates better visualization and comparison.

### 4.6 Stability of molecular feature investigation

The recently developed Nearest Template Prediction (NTP) (30) is a flexible, single-sample-based predictive method capable of cross-platform and multi-class prediction, along with confidence assessment. In the test dataset, NTP was applied to identify the three defined subtypes by utilizing the expression profiles of the signature genes. Samples with a False Discovery Rate (FDR) < 0.05 were considered successfully classified.

### 4.7 Molecular docking

Molecular docking was used to predict the binding patterns and affinities between Abemaciclib (ligand) and HOXC6(target protein). In brief, we obtained the structure of Abemaciclib from the PubChem database, which was then optimized using the MM2 force field in ChemBio3D and saved in PDB format. Based on scoring function optimization, molecular docking simulations were performed, and AutoDock Vina was used to predict the binding conformations between Savolitinib and HOXC6. The protein structure database AlphaFold was used to obtain the structure of HOXC6, which was also saved in PDB format. Subsequently, molecular docking simulations were conducted with AutoDock Vina, predicting the binding conformation of Abemaciclib and HOXC6 based on scoring function optimization. The scoring function serves as an indicator of the ligand-protein affinity, estimating the binding energy and thermodynamic stability of the protein-ligand complex. Finally, the results were visualized using PyMOL software, and the binding pose of the ligand at the protein’s active site was determined.

### 4.8 Western blot

Proteins were extracted, quantified, and separated on a 12% SDS-PAGE gel, then transferred to PVDF membranes. Membranes were blocked with skim milk and incubated withβ-actin and HOXC6 antibodies, followed by a secondary antibody. Detection was performed using an imaging system after exposure treatment.

### 4.9 Quantitative reverse transcription polymerase chain reaction

To extract total RNA of tissues, we used the RNAsimple Total RNA Kit (TIANGEN). And then the reverse-transcribed utilizing with HiScript III 1st Strand cDNA Synthesis Kit (+gDNA wiper) (Vazyme), and the NovoStart^®^ SYBR qPCR SuperMix Plus (novoprotein) was arranged for qPCR were showed in **Table 1**. The relative expression of HOXC6 was evaluated using the 2−ΔΔCt method, with GAPDH serving as the internal reference.

**Table 1:** PCR primer sequence information.

Gene	Forward	Reverse
HOXC6	ACAGACCTCAATCGCTCAGGA	AGGGGTAAATCTGGATACTGGC
GAPDH	CCAGCAAGAGCACAAGAGGAAGAG	GGTCTACATGGCAACTGTGAGGAG

### 4.10 Cell culture and HOXC6 knockout in CT26 CRC cell line

CT26 CRC cells were cultured in RPMI1640 medium supplemented with 10% fetal bovine serum (FBS) at 37°C and 5% CO2, with the medium replaced every 3 days. Cells from stable passages in the logarithmic growth phase were used for the experiments. In the knockout group, cells were infected with shRNA lentiviral particles targeting HOXC6, while the control group received lentiviral particles containing a scramble sequence.

### 4.11 CCK-8 assay

In the CCK-8 assay, CT26 cells were seeded at a density of 2×10³ cells per well in a 96-well plate and cultured overnight. Subsequently, 20 µL of CCK-8 solution (5 mg/mL) was added to each well and incubated for 4 hours. The optical density at 450 nm was measured using a microplate reader.

### 4.12 Wound healing assay

The wound healing assay was used to evaluate cell migration in CT26 cells. After the cells reached 90% confluence, a wound was made, and the wells were washed three times with PBS. Fresh serum-free medium was added, and wound closure was monitored at 0 hours and 24 hours post-wounding using an Olympus X71 microscope. The distance between the cells was measured using ImageJ. For statistical accuracy, the assay was repeated three times.

### 4.13 Colony formation assay

CT26 cells were cultured in 6-well plates under standard conditions at 37°C for two weeks. After incubation, the plates were washed with cold phosphate-buffered saline (PBS) to remove non-adherent cells. The colonies were then fixed with 4% paraformaldehyde for 15 minutes and stained with 0.1% crystal violet at room temperature. Colony formation was imaged and quantified using an optical microscope.

### 4.14 Transwell assay

The Transwell assay was used to assess cell migration. CT26 cells (4×10^4^) were placed in the upper chamber with medium containing 5% FBS, while 500 µL of medium was added to the lower chamber. After 24 hours, non-invading cells were removed, and the remaining cells were fixed with paraformaldehyde and stained with crystal violet. The number of invading cells was quantified in five random fields under a microscope.

### 4.15 Subcutaneous tumor formation experiment in mice

We conducted a subcutaneous tumor formation experiment in nude mice using CT26 cell lines with sh-NC and sh-HOXC6. First, the cells were collected by centrifugation and suspended in PBS to prepare a cell suspension, which was counted and adjusted to a concentration of 107 cells/mL. The suspension was then kept on ice and mixed thoroughly before injection. Next, the right side of the rib cage of the nude mice was disinfected, and 0.2 mL of the cell suspension was injected using a 1 mL syringe. The nude mice were housed separately according to different experimental groups, and their initial body weights were recorded on the day of injection. From the day of injection, the body weight of the nude mice and the length and width of the subcutaneous tumor were recorded every 2 days for a total observation period of 15 days. On day 15 of the experiment, we recorded the body weight and tumor size again, after which the mice were euthanized, and photographs were taken for documentation. Finally, the nude mice were dissected, and the tumor tissues were extracted for weighing and photography for subsequent analysis.

### 4.16 *In vivo* antitumor experiment with savolitinib

We conducted a subcutaneous tumor formation experiment in nude mice using the CT26 cell line, following the same steps as described above. Three days prior to tumor implantation and every three days thereafter, Savolitinib and its control DMSO were injected around the tumor, with a dosage of 10 mg/kg.

### 4.17 Statistical analysis

All data processing, visualization, and statistical analyses were performed using R software. The correlation between two continuous variables was assessed using Spearman’s correlation coefficient. Initially, normality tests were performed on the datasets. For normally distributed data with equal variances, Student’s t-test and one-way analysis of variance (ANOVA) were used to compare differences between two or more groups. For non-normally distributed data or data that did not meet the homogeneity of variance assumption, the Wilcoxon test and Kruskal-Wallis test were used for comparisons within two or more groups, respectively. Chi-square tests were used to analyze categorical variables. p-values were adjusted for false discovery rate (FDR), particularly when performing multiple pairwise comparisons. To validate the results, the experiments were performed in triplicate to confirm their reproducibility.

## Data Availability

The original contributions presented in the study are included in the article/supplementary material. Further inquiries can be directed to the corresponding author.
